# Perinatal fluoxetine treatment and dams’ early life stress history have opposite effects on aggressive behavior while having little impact on sexual behavior of male rat offspring

**DOI:** 10.1007/s00213-020-05535-7

**Published:** 2020-07-17

**Authors:** Danielle J. Houwing, Diana C. Esquivel-Franco, Anouschka S. Ramsteijn, Kirsten Schuttel, Eline L. Struik, Chantal Arling, Sietse F. de Boer, Jocelien D. A. Olivier

**Affiliations:** Department of Neurobiology, GELIFES, University of Groningen, Nijenborgh 7, 9747 AG Groningen, the Netherlands

**Keywords:** SSRI, Development, Serotonin transporter, Early life stress, Behavior, Offspring, Depression

## Abstract

**Rationale:**

Many depressed women continue antidepressant treatment during pregnancy. Selective serotonin reuptake inhibitor (SSRI) treatment during pregnancy increases the risk for abnormal social development of the child, including increased aggressive or defiant behavior, with unknown effects on sexual behavior.

**Objectives:**

Our aim was to investigate the effects of perinatal SSRI treatment and maternal depression, both separately and combined, on aggressive and sexual behavior in male rat offspring.

**Methods:**

Heterozygous serotonin transporter (SERT^±^ ) knockout dams exposed to early life stress (ELSD) were used as an animal model of maternal depression. Early life stress consisted of separating litters from their mother for 6 h a day on postnatal day (PND)2–15, resulting in a depressive-like phenotype in adulthood. Depressive-like dams were treated with fluoxetine (FLX, 10 mg/kg) or vehicle throughout pregnancy and lactation (gestational day 1 until PND 21). Male offspring were tested for aggressive and sexual behavior in adulthood. As lifelong reductions in SERT expression are known to alter behavioral outcome, offspring with normal (SERT^+/+^) and reduced (SERT^±^ ) SERT expression were assessed.

**Results:**

Perinatal FLX treatment reduced offensive behavior and the number of animals attacking and increased the latency to attack, especially in SERT^+/+^ offspring. Perinatal FLX treatment reduced the mounting frequency in SERT^+/+^ offspring. ELSD increased offensive behavior, without affecting sexual behavior in SERT^±^ offspring.

**Conclusions:**

Overall, our research demonstrates that perinatal FLX treatment and ELSD have opposite effects on aggressive behavior, with little impact on sexual behavior of male offspring.

## Introduction

Many women who initiate or continue antidepressant treatment during pregnancy are prescribed selective serotonin reuptake inhibitors (SSRIs), as they are considered relatively safe for both mother and child (Gentile 2005). However, SSRIs are able to cross the placenta and are excreted into breast milk, thus reaching the developing child (Heikkinen et al. 2003; Noorlander et al. 2008). SSRIs act on the serotonergic system by blocking the serotonin transporter, resulting in sustained higher extracellular serotonin (5-HT) levels and increased serotonergic neurotransmission (Pierz and Thase 2014). In the developing brain, 5-HT acts as a neurotrophic factor, regulating a wide variety of neurodevelopmental processes including neurogenesis, cell division, differentiation and migration, neuroapoptosis, and synaptic plasticity (Azmitia 2001; Gaspar et al. 2003; Sodhi and Sanders-Bush 2003). Therefore, it has been suggested that perinatal SSRI exposure has the potential to influence serotonergic functioning and subsequently alter behavioral development of the child. Indeed, exposure to SSRIs has been linked to abnormal development of social behaviors (reviewed by Gemmel et al. 2018a). Clinical studies show that children from mothers treated with SSRIs during pregnancy have an increased risk to show more externalizing behaviors such as aggression or defiant behavior (Oberlander et al. 2007) and more internalizing behaviors such as anxiety, depression, and social withdrawal (Hanley et al. 2015; Oberlander et al. 2010). Furthermore, there is an ongoing discussion about whether SSRI treatment increases the risk for the child to develop autism spectrum disorder, as effects often disappear when controlling for maternal illness (Brown et al. 2017; Kaplan et al. 2017; Zhou et al. 2018). In preclinical studies, it has been shown that developmental SSRI exposure affects various social behaviors in rodents, including aggressive and sexual behavior. For instance, treating rat dams with fluoxetine (FLX) during the prenatal period increased the number of fighting bouts in adult male offspring, without affecting attack latency (Singh et al. 1998). Treating mice dams with FLX during the prenatal and early postnatal period resulted in an increased number of offspring attacking intruder mice, even though they showed a similar amount of aggressive behavior compared with control mice (Kiryanova and Dyck 2014). Concerning sexual behavior, offspring from mice dams treated with FLX from conception until weaning showed reduced sexual incentive motivation, without affecting copulatory behaviors (Gouvêa et al. 2008). In rats, a decrease in copulatory behaviors including number of mounts, intromission, and ejaculations has been found after exposure to the SSRI citalopram during the early postnatal period (Harris et al. 2012; Maciag et al. 2006; Rodriguez-Porcel et al. 2011). These data indicate that perinatal SSRI exposure increases aggressive behavior, while decreasing sexual behavior. Interestingly, similar neural structures are involved in inter-male aggression and reproductive behavior (Anderson 2012). Even though the pathways of reproductive and offensive behaviors are shared, exposure to perinatal FLX appears to have opposite effects on aggressive and sexual behavior in male rodent offspring. Since it is difficult to discern the effects of maternal depression and SSRI treatment in humans, animal models of maternal stress are of great help to separate the effects of maternal depression and SSRI treatment during pregnancy on offspring behavior. From a translational perspective, studying the effects of perinatal SSRI exposure in an animal model of depression is highly relevant, as usually depressed and not healthy women take SSRIs. In rodents, stress during the prenatal period has been associated with reduced aggression in male offspring (Kinsley and Svare 1986; Patin et al. 2005). In contrast, stressors during the early postnatal period, such as maternal separation or social deprivation, have been found to increase aggressive behavior in rodents (Sandi and Haller 2015). Besides aggressive behavior, it has also been shown that both prenatal and early postnatal stress in rodents reduce male sexual behavior, especially the number of copulatory behaviors (Masterpasqua et al. 1976; Rhees and Fleming 1981), and result in less animals showing at least one intromission or ejaculation response (Hernández-Arteaga et al. 2016; Rhees et al. 2001). The number of preclinical studies combining the effects of maternal stress and SSRI exposure during the perinatal period to investigate offspring neurodevelopment is increasing but still limited when it comes to aggressive and sexual behavior. Kiryanova et al. (2016) studied the effects of FLX treatment in mouse dams exposed to prenatal chronic unpredictable tress. They found that perinatal FLX treatment increased levels of aggressive behavior, while prenatal stress reduced levels of aggressive behavior in male offspring mice (Kiryanova et al. 2016). Interestingly, when FLX treatment was administered in dams exposed to early life stress, offspring displayed normal aggressive behavior. When it comes to sexual behavior, Rayen et al. (2013) found that FLX treatment during lactation reduced the number of intromissions and resulted in a longer latency to the first intromission and ejaculation in male offspring. However, prenatal restraint stress and the combination of prenatal stress and FLX treatment did not affect copulatory behavior of male offspring (Rayen et al. 2013). While these studies use an animal model of prenatal stress, pregestational stress might be more clinically relevant, as many women cope with a depression long before they become pregnant. Therefore, we investigated the effects of perinatal FLX treatment on aggressive and sexual behavior in male offspring using an animal model of pregestational maternal depression. Our animal model of maternal depression consists of heterozygous serotonin transporter knockout (SERT^±^ ) dams that as pups have been exposed to early life stress (early life stress in dams, ELSD), resulting in depressive-like behavior (Houwing et al. 2019a). As reduced SERT expression has been associated with increased vulnerability to develop anxiety and depressive-like behavior after stressful life events (Caspi et al. 2003), but also with poorer response to FLX treatment in adults (Stevenson 2018), we expected offspring SERT genotype to interact with FLX and ELSD. Furthermore, as it has been shown that perinatal SSRI treatment can increase aggressive behavior in rodent offspring (Kiryanova et al. 2016; Kiryanova and Dyck 2014; Singh et al. 1998), we expected a similar increase in aggression toward an intruder after perinatal FLX treatment. In addition, we expected ELSD to reduce offspring aggressive behavior (Kinsley and Svare 1986; Kiryanova et al. 2016; Patin et al. 2005). When offspring is exposed to perinatal FLX and ELSD, we expect aggressive behavior in offspring to be normal (Kiryanova et al. 2016). Furthermore, since multiple studies show reduced sexual behavior after both SSRI exposure (Gouvêa et al. 2008; Maciag et al. 2006; Rayen et al. 2013) and prenatal stress (Hernández-Arteaga et al. 2016; Masterpasqua et al. 1976; Rhees and Fleming 1981), we similarly expected perinatal FLX treatment and ELSD, independently as well as combined, to reduce sexual behavior in the offspring.

## Materials and methods

### Animals

Wistar rats were maintained on a reversed 12-h light/dark cycle (lights off at 11:00 a.m.) with ad libitum access to food (RMH-B, AB Diets; Woerden, the Netherlands) and tap water. A wooden gnawing stick (10 × 2 × 2 cm) and nesting material (Enviro-dri®) was provided for environmental enrichment. Animals were housed in Makrolon type 3 cages (38.2 × 22.0 × 15.0 cm) during individual housing or Makrolon type 4 cages (55.6 × 33.4 × 19.5 cm) during social housing. All breeding occurred in our own facility. All experimental procedures were approved by the Groningen University Committee of Animal experiments (DEC 6936A).

### Early life stress in dams

Serotonin transporter knockout rats (Slc6a41Hubr) were bred crossing SERT^+/−^ females with SERT^±^ males, resulting in pups of three genotypes (SERT^+/+^, SERT^±^ , and SERT^−/−^). These pups (future dams) were randomly assigned to either the control group (CTR) or the early life stress group (ELS). ELS consisted of maternally separating both male and female pups of all SERT genotypes as a whole litter for 6 h a day from postnatal day (PND)2–15. CTR pups were taken away during this period for 15 min and handled briefly. At PND21, pups were weaned and socially housed with same-treated, same sex pups from different litters. When adult, SERT^±^ females exposed to ELS show depressive-like behavior (Houwing et al. 2019a) and were therefore used as an animal model for maternal depression.

### Perinatal fluoxetine treatment

In total, 85 (33 CTR and 52 ELS) female SERT^±^ and 47 male SERT^+/+^ rats were used for breeding. Female estrus was determined by measuring vaginal wall impedance (model MK-11, Muromachi, Tokyo, Japan) and followed by housing the receptive female together with a male for 24 h (gestational day 0, G0). CTR and ELS SERT^±^ females were randomly assigned to the vehicle-treated (VEH) or fluoxetine-treated (FLX) group. Using oral gavage, dams were treated daily with VEH (methylcellulose 1%, Sigma-Aldrich Chemie BV, Zwijndrecht, the Netherlands) or FLX (10 mg/kg; Pharmachemie BV, the Netherlands) injected at a volume of 5 mL/kg from G0 until weaning of the pups at PND21. For oral gavage, flexible PVC feeding tubes (Vygon, Valkenswaard, the Netherlands) were used without restraining the animals, thus minimizing stress. To determine the exact dosing volume, dams were weighed daily. Four groups of dams were used: (1) CTR dams + VEH treatment (CTR-VEH) (*n* = 11), (2) CTR dams + FLX treatment (CTR-FLX) (*n* = 22), (3) ELS in dams + VEH treatment (ELSD-VEH) (*n* = 15), and (4) ELS in dams + FLX treatment (ELSD-FLX) (*n* = 37). Dams were checked twice a day for pup delivery. Pregnancy outcomes of the dams and litter characteristics have been reported elsewhere (Houwing et al. 2019b). On PND21, pups were weaned, and ears were punched for individual recognition and genotyping (El Aidy et al. 2017). SERT^+/+^ and SERT^±^ offspring were housed with 3–5 same-treated, same-sex animals from different litters while mixing genotypes. Due to unexpected high mortality rates in dams and offspring from FLX groups (Houwing et al. 2019b), offspring from three (aggressive behavior) or even four (sexual behavior) batches were needed to complete this study. In addition, because of the reduced survival of FLX pups, 4.7% of the litters (batch 1 to 3) up to five pups per litter were used. No litter effects were found.

### Behavioral testing

Both SERT^+/+^ and SERT^±^ male offspring were tested for aggressive and sexual behavior during adulthood. Different batches of males were used for aggressive and sexual behavior. Males used for aggressive behavior were previously tested for juvenile play (4–5 weeks of age) and social interaction (10–12 weeks of age) (Houwing et al. 2019b). Males used for sexual behavior were previously tested for affective behavior, including the open field test (16 weeks of age), the forced swim test (26 weeks of age), and the sucrose preference test (27–30 weeks of age) (Houwing et al., in preparation). Testing occurred between 12:00 p.m. and 5:00 p.m. during the active dark phase of the animals. Behavior was manually scored and analyzed by one observer blind to treatment using The Observer XT version 11.0 (Noldus Information Technology B.V., Wageningen, The Netherlands).

#### Aggressive behavior

Male (14–16 weeks old) offspring (SERT^+/+^: CTR-VEH *n* = 11, CTR-FLX *n* = 11, ELSD-VEH *n* = 11, ELSD-FLX *n* = 10. SERT^±^ : CTR-VEH *n* = 11, CTR-FLX *n* = 11, ELSD-VEH *n* = 9, ELSD-FLX *n* = 11) were tested for aggressive behavior in the resident-intruder test (Koolhaas et al. 2013). Male residents were paired with an adult non-experimental SERT^+/+^ female and housed in a large cage (80 × 55 × 50 cm) for 1 week to habituate and establish territorial behavior. Females were oviduct ligated 2 weeks before the habituation period, to prevent pregnancy and the development of maternal aggression while keeping hormonal regulation intact. Pairs were housed with a polycarbonate tunnel and 2 wooden gnawing sticks (10 × 2 × 2 cm) for enrichment, while food (RMH-B, AB Diets; Woerden, the Netherlands), and tap water were present ad libitum. Cages were covered with a 2-cm layer of Aspen wood chip bedding and were not cleaned during the habituation and testing period. Females and enrichment were removed at least 1 h before testing, and testing occurred under dim light conditions (10 lx). Residents received three training sessions followed by a test session on consecutive days. During training, an unfamiliar non-experimental male SERT^+/+^ rat (intruder) was introduced into the resident’s cage, and the latency to attack the intruder by biting was observed. After the first biting attack, or when no attack occurred within 10 min, the intruder was removed. Residents and intruders were weighed prior to habituation to ensure that intruders were lighter than residents. Intruders were only used once a day, and each resident received an unfamiliar intruder for each training and test session. During the test session, aggressive behavior of the resident was recorded on video for 10 min after the first attack. If residents did not attack the intruder, the first 10 min of the video were scored. The frequency and duration of the following behaviors of the resident were manually scored from the video recordings: (1) offensive behavior (lateral threat, upright posture, clinch attack, keep down, and chasing), (2) social behavior (moving toward/following, social sniffing, social grooming), (3) defensive behavior (keep off and submissive behavior), and (4) non-social behavior (non-social exploration, rearing, inactivity, and self-grooming). Furthermore, the latency to attack the intruder was scored. In addition, we calculated the proportion of animals attacking the intruder during the test session. Behavioral measures were converted into percentage of total time spent on the behavior.

#### Sexual behavior

Male (32 weeks old) offspring (SERT^+/+^: CTR-VEH *n* = 10, CTR-FLX *n* = 10, ELSD-VEH *n* = 11, ELSD-FLX *n* = 10, SERT^±^ : CTR-VEH *n* = 10, CTR-FLX *n* = 10, ELSD-VEH *n* = 11, ELSD-FLX *n* = 11) were assessed for sexual behavior in 45 × 30 × 50 cm Phenotyper cages (Noldus Information Technology B.V., Wageningen, The Netherlands) under red light conditions. Cages were covered with a 2-cm layer of Aspen wood chip bedding and were not cleaned during the testing period. Adult female SERT^+/+^ rats were used as stimulus animals, and estrous was induced with a single s.c. injection of 50 μg estradiol benzoate (dissolved in sesame oil saturated with lecithin) 36–42 h prior to testing (Olivier et al. 2011). First, males were allowed to habituate to the test cage for 10 min. Subsequently, a receptive female was introduced, and sexual behavior was recorded on video for 30 min. To ensure the male was able to interact with a receptive and willing female, female behavior was observed within the first 5 min of the test. If she repeatedly rejected the male (e.g., kicking, turning around to prevent mounting), she was replaced. Training of sexual behavior occurred once a week for 6 consecutive weeks. Sexual behavior was recorded on video tape in week 7, when the animals were sexually experienced. The frequency and latency of the following male copulatory behaviors were manually scored from video: mounts, intromissions, and ejaculations. Mounting and intromission frequency of the first ejaculatory series (frequency up until the first ejaculation) were used. Copulatory efficiency was calculated by determining the intromission ratio: number of intromissions/(number of intromissions + number of mounts)*100.

### Statistical analysis

Data was analyzed using the Statistical Package for the Social Sciences (SPSS) software version 22 (SPPS Inc., IBM SPSS Statistics, Chicago). Upon non-parametric distribution, data was transformed to approach parametric distribution for use in statistical analysis. For behavior in the resident-intruder test, offensive and defensive behaviors were log transformed. Regarding sexual behavior, a square root transformation was performed for mounting, intromission, and ejaculation frequency. In addition, latency of copulatory behaviors was log transformed. All other behavioral parameters approached parametric distribution.For both aggressive and sexual behavior, a three-way ANOVA was performed to determine main and/or interaction effects of FLX, ELSD, and genotype. There were no significant 3-way interactions found for any of the behavioral parameters analyzed (*p* > .05). When found, two-way interactions and main effects are described in the results. For the proportion of animals attacking, a *χ*^2^ analysis was performed. Upon significant main and/or interaction effects, post hoc testing was performed using Fisher’s LSD to correct for multiple comparisons. All statistics were two-tailed with values of *p* ≤ .05 being considered significant. When adding litter as a factor in our ANOVA, no significant interactions between litter and other factors were found. All data and all figures are presented as mean ± standard error of the mean (SEM). Outliers were not removed, and individual data points are shown in each figure.

## Results

### Aggressive behavior

**Fig. 1:**
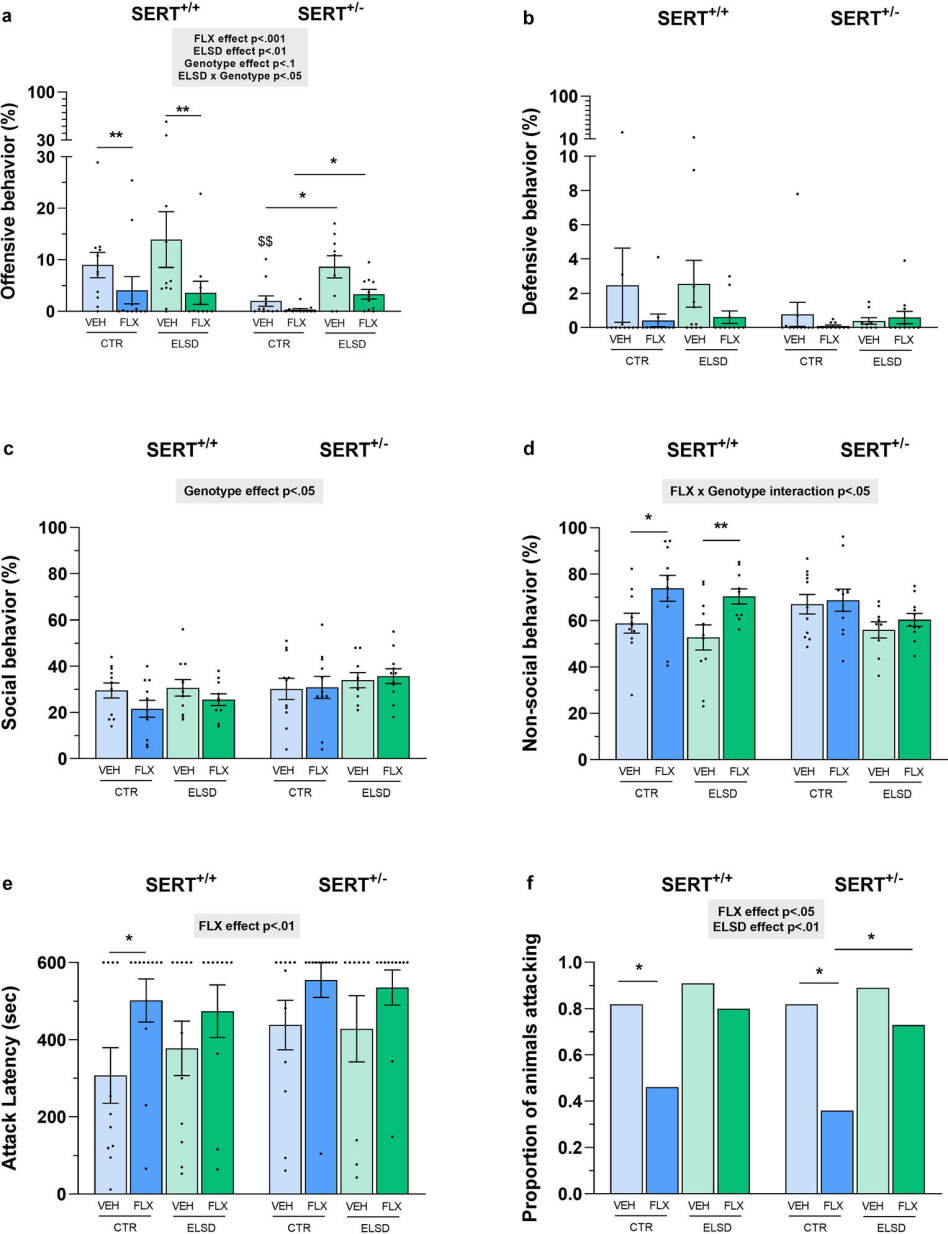
Effects of perinatal FLX treatment and ELSD on various behaviors of adult male offspring in the resident intruder test. Shown are offensive behavior (**a**), defensive behavior (**b**), social behavior (**c**), non-social behavior (**d**), attack latency (**e**), and proportion of animals attacking (**f**) during the test session. Figures show mean ±SEM. *p ≤ .05, **p < .01, $$ *p* < .01 (vs. SERT^+/+^ CTR-VEH). *n* = 9–12 per group

Effects of perinatal FLX treatment and ELSD on aggressive behavior in SERT^+/+^ and SERT^±^ male offspring were assessed using the resident intruder test. Perinatal FLX treatment significantly reduced offensive behavior in the offspring, regardless of ELSD and genotype (F_(1,77)_ = 17.686, *p* < .001, Fig. 1a). Post hoc testing revealed that SERT^+/+^ CTR-FLX offspring showed less offensive behavior compared with SERT^+/+^ CTR-VEH offspring (*p* < .01, Fig. 1a). Similarly, SERT^+/+^ ELSD-FLX offspring displayed less offensive behavior compared with SERT^+/+^ ELSD-VEH offspring (*p* < .01, Fig. 1a). Furthermore, ELSD significantly increased offensive behavior in the offspring, regardless of perinatal FLX treatment and genotype (F_(1,77)_ = 7.389, *p* < .01, Fig. 1a). In addition, an interaction between ELSD and genotype was found (F_(1,77)_ = 4.786, *p* < .05), with both SERT^±^ ELSD-VEH and SERT^±^ ELSD-FLX offspring showing significantly increased offensive behavior compared with SERT^±^ offspring from same**-**treated CTR dams (CTR-VEH vs. ELSD-VEH: *p* < .05, CTR-FLX vs. ELSD-FLX: *p* < .05), while SERT^+/+^ offspring were not affected (Fig. 1a). Also, a tendency toward a main effect of genotype was found with SERT^±^ offspring showing less offensive behavior compared with SERT^+/+^ offspring (F_(1,77)_ = 3.264, *p* = .08, Fig. 1a). More specifically, post hoc testing revealed that SERT^±^ CTR-VEH offspring showed less offensive behavior compared with SERT^+/+^ CTR-VEH offspring (*p* < .01, Fig. 1a). Defensive behavior was not affected by ELSD, perinatal FLX treatment**,** or offspring genotype (*p* > .05, Fig. 1b). When looking into non-aggressive social behavior, a significant main effect of genotype was found with SERT^±^ offspring showing increased social behavior compared with SERT^+/+^ offspring, regardless of perinatal FLX treatment and ELSD (F_(1,77)_ = 4.901, *p* < .05, Fig. 1c). Regarding non-social behavior, a significant interaction between perinatal FLX treatment and genotype was found (F_(1,77)_ = 4.578, *p* < .05, Fig. 1d). Both SERT^+/+^ CTR-FLX and SERT^+/+^ ELSD-FLX offspring showed increased non-social behavior compared with VEH**-**treated counterparts (CTR-FLX vs. CTR-VEH: *p* < .05, ELSD-FLX vs. ELSD-VEH: *p* < .01)**,** while perinatal FLX treatment did not affect non-social behavior in SERT^±^ offspring (Fig. 1d). For the attack latency, there was a significant main effect found of perinatal FLX treatment, with perinatal FLX treatment increasing the latency to attack the intruder, regardless of ELSD and genotype (F_(1,77)_ = 8.101, *p* < .01, Fig. 1e). Post hoc analysis showed that SERT^+/+^ CTR-FLX offspring had a higher latency to attack the intruder than SERT^+/+^ CTR-VEH offspring (*p* < .05, Fig. **1**e). Regarding the proportion of animals attacking the intruder, a significant main effect of perinatal FLX treatment was found, with a smaller proportion of animals attacking, regardless of ELSD and genotype (*χ*^2^_(1,85)_ = 7.973, *p* < .01, Fig. 1f). Also, a significant main effect of ELSD was found, with a higher proportion of animals attacking the intruder, regardless of perinatal FLX treatment and genotype (*χ*^2^_(1,85)_ = 4.870, *p* < .05, Fig. 1f). Post hoc testing revealed that a smaller proportion of SERT^+/+^ CTR-FLX offspring attacked the intruder than SERT^+/+^ CTR-VEH offspring (*p* = .05, Fig. **1**f). Also, a significant smaller proportion of SERT^±^ CTR-FLX offspring attacked the intruder compared with both SERT^±^ CTR-VEH (*p* < .05) and SERT^±^ ELSD-FLX offspring (*p* = .05).

### Sexual behavior

**Fig. 2:**
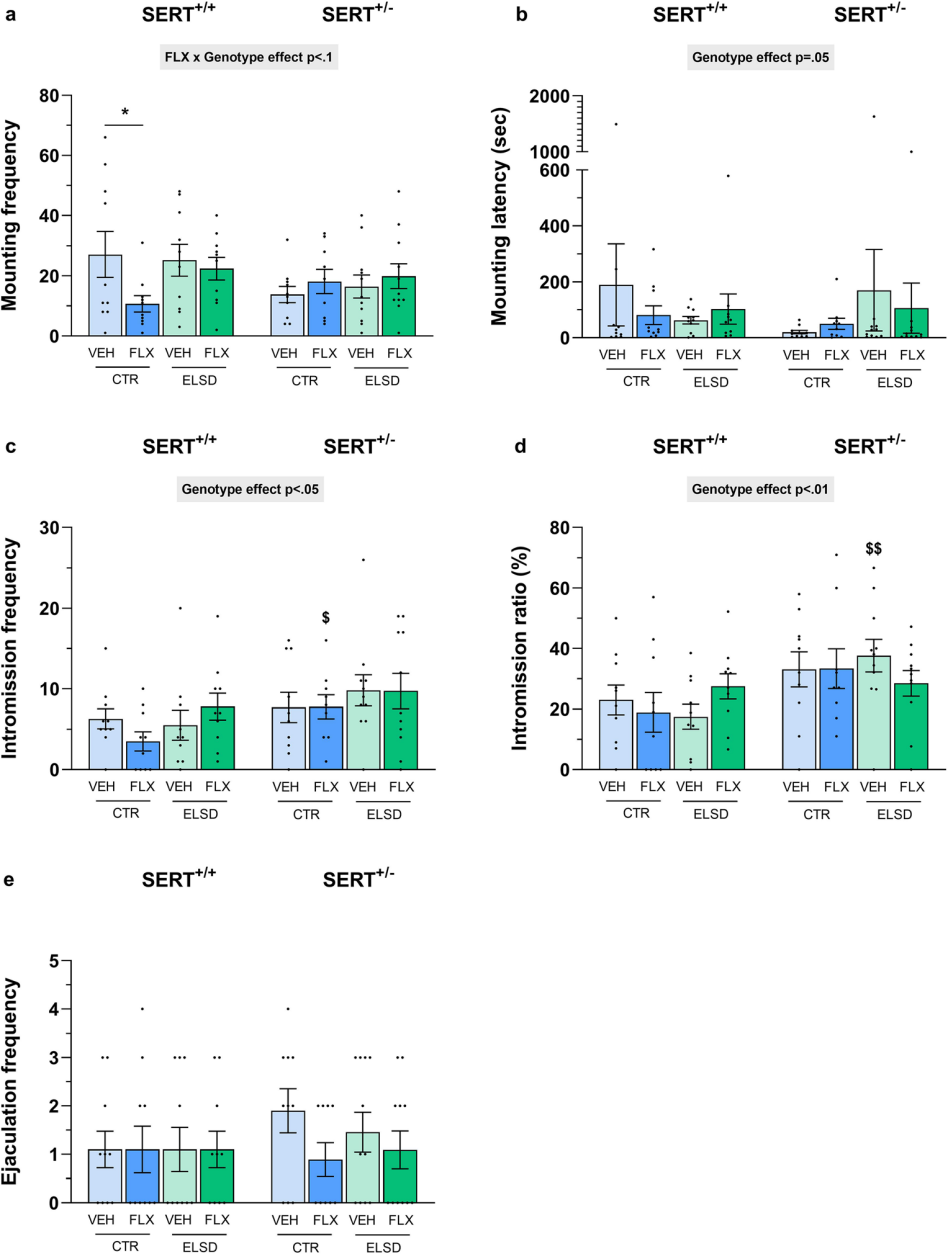
Effects of perinatal FLX treatment and ELSD on male offspring copulatory behaviors. Shown are the mounting frequency (**a**), mounting latency (**b**), intromission frequency (**c**), intromission ratio (**d**), and the ejaculation frequency (**e**). Figures show mean ±SEM. *p ≤ 0.05, $ *p* ≤ 0.05 (vs. SERT^+/+^), $$ *p* < 0.01 (vs. SERT^+/+^). *n* = 10–11 per group

Effects of perinatal FLX treatment and ELSD on male copulatory behaviors in SERT^+/+^ and SERT^±^ offspring were assessed after 6 weeks of training, when animals were sexually experienced. A tendency toward an interaction between perinatal FLX treatment and genotype was found for mounting frequency (F_(1,75)_ = 3.671, *p* = .06), with post hoc testing revealing that SERT^+/+^ CTR-FLX offspring showed less mounting behavior compared with SERT^+/+^ CTR-VEH offspring (*p* < .05, Fig. 2a). When looking at the latency until the first mount, a significant main effect of genotype was found, with SERT^±^ offspring showing a reduced mounting latency compared with SERT^+/+^ offspring, regardless of perinatal FLX treatment and ELSD (F_(1,75)_ = 3.923, *p* = .05, Fig. 2b). However, post hoc comparisons show no genotype differences between offspring groups. For the number of intromissions, again a significant main effect of genotype was found, with SERT^±^ offspring displaying an increased number of intromissions compared with SERT^+/+^ offspring, regardless of perinatal FLX treatment and ELSD (F_(1,75)_ = 5.702, *p* < .05, Fig. 2c). More specifically, post hoc analysis showed that SERT^±^ CTR-FLX offspring performed more intromissions than SERT^+/+^ CTR-FLX offspring (*p* < .05, Fig. 2c). Also, for the intromission ratio, a significant main effect of genotype was found, with SERT^±^ offspring showing a higher intromission ratio than SERT^+/+^ offspring, regardless of perinatal FLX treatment and ELSD (F_(1,75)_ = 9.924, *p* < .01, Fig. 2d). Post hoc analysis revealed that SERT^±^ ELSD-VEH offspring had a higher intromission ratio than SERT^+/+^ ELSD-VEH offspring, suggesting higher copulatory efficiency (Fig. 2d). No other significant main and/or interaction effects were found for the ejaculation frequency (Fig. 2e) and other behavioral parameters for copulatory behavior. Between batches, animals significantly differed in their sexual performance (ejaculation frequency: F_(3,82)_ = 14.927, *p* < .001). With an average ejaculation frequency below 1, animals in the second (0.53 ± 0.48), third (0.90 ± 0.21), and fourth (0.64 ± 0.29) batch were poor sexual performers and differed significantly from the first batch (2.48 ± 0.20, *p* < .001), where animals performed normally.

## Discussion

In this study, we sought to explore the effects of perinatal FLX treatment and dams’ early life stress history, both separately and combined, on aggressive and sexual behavior in adult SERT^+/+^ and SERT^±^ male offspring. Our main findings demonstrate that perinatal FLX treatment lowered aggressive behavior, especially in SERT^+/+^ offspring, while early life stress in dams (ELSD) heightened aggressive behavior in SERT^±^ offspring. Perinatal FLX treatment caused mild effects on sexual behavior of SERT^+/+^ offspring, while ELSD did not affect sexual behavior. Finally, we found genotype**-**specific effects. SERT^±^ offspring showed less offensive but more sexual activity compared with SERT^+/+^ rats. Altogether**,** these data show that both perinatal FLX treatment and ELSD genotype dependently affect social behavior in the offspring.

### Effects of perinatal FLX treatment on offspring aggressive behavior

Against our expectations, FLX treatment in healthy dams reduced aggressive behavior in SERT^+/+^ offspring. This was reflected in less time spent in offensive behavior, an increased attack latency, and a smaller proportion of animals attacking the intruder. Our findings contradict both clinical and preclinical data, where perinatal SSRI treatment has been associated with an increase in aggressive behavior. In humans, SSRI treatment during pregnancy has been associated with increased externalizing behaviors such as aggression or defiant behavior in 4-year-olds (Oberlander et al. 2007). Similarly, preclinical studies show that perinatal FLX treatment has been associated with an increase in male aggressive behavior during adulthood. FLX treatment during the last week of pregnancy increased the number of foot shock-induced aggressive fighting bouts in adolescent rat offspring (Singh et al. 1998). FLX treatment during the entire pregnancy increased the number and duration of aggressive attacks in adult male mice (Svirsky et al. 2016). In addition, Kiryanova and Dyck (2014) found that perinatal FLX treatment from G15–PND21 increased the proportion of mice attacking an intruder, while other parameters of aggressive behavior were unaffected. Related to the reduced aggression levels we found in the present study, Yu et al. (2014) showed that male mice exposed to FLX during PND2–PND21 displayed reduced aggressive behavior toward an intruder, even failing to display some characteristic aggressive behaviors such as biting and tail rattling. Furthermore, Lisboa et al. (2007) exposed male mice offspring to FLX throughout pregnancy and lactation (G0–PND21), similar to our study, and found a trend toward an increased attack latency but no effects on number of attacks or attack duration in adult male offspring. All taken together, it seems that prenatal FLX treatment increases offspring aggressive behavior, while postnatal FLX exposure reduces aggressive behavior. However, when FLX is given during both the pre- and postnatal period, results on aggressive behavior in the offspring are inconsistent. One possible explanation for the found reduction in offspring aggressive behavior is that our animals are less motivated to participate in social interactions. We previously found that the animals used in this study engage less in juvenile social play behavior and adult social interactions (Houwing et al. 2019b). Indeed, in the present study, we found that FLX-exposed offspring displayed more non-social behaviors, such as non-social exploration. At the same time, FLX-exposed offspring spent a similar amount of time resting, indicating that FLX-exposed offspring were not simply less active than VEH-exposed offspring (data not shown).

Interestingly, reduced aggressive behavior after developmental FLX exposure corresponds with findings in SERT^−/−^ rodents, who similarly display reduced levels of aggression. Both SSRI exposure and diminished SERT expression result in elevated extracellular serotonin levels. Whereas SERT^−/−^ rodents have chronically elevated 5-HT levels and display reduced aggressive behavior compared with SERT^+/+^ rodents, SERT^±^ rodents have similar extracellular 5-HT levels to SERT^+/+^ (reviewed by Houwing et al. 2017). Interestingly, we observed reduced offensive behavior in SERT^±^ CTR-VEH offspring when compared with SERT^+/+^ offspring, while it is often reported that SERT^+/+^ and SERT^±^ rodents show no differences in behavior. Perhaps these rats function similar under normal conditions but may respond differently under stressful situations. Overall, we found reduced aggressive behavior when animals are exposed to FLX. Discrepancies between studies in aggressive behavior could be attributed to several factors, including the variation in behavioral tests to measure aggressive behavior, species used, FLX dose, and treatment period (pre- versus postnatal) and treatment duration (days versus weeks).

### Effects of perinatal FLX treatment on offspring sexual behavior

Regarding sexual behavior, our findings demonstrated that male CTR offspring, but not ELSD offspring, exposed to perinatal FLX show reduced mounting behavior, specifically in SERT^+/+^ offspring. However, other copulatory behaviors such as intromissions, ejaculations**,** and the intromission ratio were not affected, suggesting that sexual motivation is only slightly reduced, while leaving sexual performance intact. In humans**,** not much is known about SSRI treatment during pregnancy and lactation and the effects it can have on offspring sexual behavior later in life, but preclinical studies found that early SSRI exposure can lead to reduced copulatory behaviors in rodents. For example, when rat pups are treated with the SSRI citalopram from PND8**–**PND21, the number of mounts, intromissions**,** and ejaculations are reduced in both naïve and sexually experienced male rats (Harris et al. 2012; Maciag et al. 2006; Rodriguez-Porcel et al. 2011). Similarly, FLX exposure during the postnatal period reduced sexual motivation and performance in adult male rats (Rayen et al. 2013; Rodriguez-Porcel et al. 2011). However, when animals were exposed to FLX during the prenatal period, or throughout the pre- and postnatal period, male copulatory behavior was unaffected in mice (Gouvêa et al. 2008) and rats (Cagiano et al. 2008; Olivier et al. 2011; Vieira et al. 2013). All in all, the present study shows that perinatal FLX exposure did not affect overall sexual performance, although a small decrease was found in sexual motivation.

### Effects of ELSD on offspring aggressive and sexual behavior

Against our expectations, ELSD increased aggressive behavior in male SERT^±^ offspring, which was reflected in increased offensive behavior toward an intruder. However, when these pups were also exposed to FLX, aggression levels were comparable with the control animals. Our findings contradict previous literature where prenatal stress reduced aggressive behavior in male offspring (Kinsley and Svare 1986; Kiryanova et al. 2016; Patin et al. 2005). While prenatal stress reduces aggressive behavior in offspring, stressors during the early postnatal period, such as maternal separation, have the potential to increase inter-male aggression in rats (Veenema et al. 2006) and maternal aggression in mice (Veenema et al. 2007). Although the offspring in the present study were not exposed to maternal separation, their mothers were exposed to maternal separation. Because transgenerational effects of early life stress have been reported in both humans (Esteves et al. 2019) and rodents (Weiss et al. 2011), our results in the offspring may be the result of transgenerational effects of early life stress in our dams. One of the mechanisms responsible for the potential transgenerational effects on aggressive behavior is through physiological changes in the dam. Both prenatal stress (Rakers et al. 2017) and maternal separation can induce maternal cortisol (or corticosterone (CORT) in rodents) release which can be transferred to the fetus via the placenta to induce changes in the fetal hypothalamic-pituitary-adrenal axis and subsequent behavioral alterations later in life. In fact, maternal separation for 3 h a day on PND1–14 can increase basal CORT levels and other regulators of the HPA-axis such as hypothalamic CRF mRNA levels in juvenile (Veenema and Neumann 2009) and adult (Plotsky and Meaney 1993) male rats. However, 6 h of maternal separation from PND2–15 in our dams did not affect basal CORT levels prior to breeding (Houwing et al. 2019a) and was not measured during pregnancy or in the offspring. Even so, a depressive-like phenotype during pregnancy is likely to induce elevated stress levels in the dams, and maternal-fetal stress transfer is likely to occur (Brummelte and Galea 2010), but we do not know whether maternal or fetal CORT levels are correlated with aggression levels in the offspring. Whether the dams exposed to early life stress indeed show more maternal aggression and if these effects are transferred to the offspring remain to be further investigated. Another possibility for the increased aggression found in our offspring is due to altered maternal care of the dam. Prenatal stress can either increase (Rayen et al. 2011), reduce (Smith et al. 2004), or have no effect on maternal care of the dam (Gemmel et al. 2018b; Kiryanova et al. 2016; Pawluski et al. 2012a, b). Predictive maternal separation can increase maternal care of the dam (Franklin et al. 2010), and there is evidence for the behavioral transmission of maternal care behavior from dams to female offspring (reviewed by Champagne 2008). Whether the observed increase in aggressive behavior in offspring of mothers exposed to early life stress are associated with altered maternal care levels remains to be investigated. Regarding sexual behavior, no effects of ELSD was found on copulatory behaviors of male offspring. These findings are opposed to previous studies that have repeatedly found prenatal stress to impair male sexual behavior (Gerardin et al. 2005; Hernández-Arteaga et al. 2016; Masterpasqua et al. 1976; Wang et al. 2006) but are similar to Rayen and colleagues who found no effects of prenatal stress on male (Rayen et al. 2013) and female (Rayen et al. 2014) sexual performance. To our knowledge, the effects of ELSD on male sexual behavior in the next generation have not been documented. Clearly, more research is needed on the transgenerational effects of ELSD on sexual behavior performance of male offspring.

### Effects of offspring SERT genotype and its interaction with perinatal FLX treatment and ELSD

Our results demonstrated that SERT^±^ males showed reduced offensive behavior and increased non-aggressive social behavior compared with SERT^+/+^ offspring. Furthermore, SERT^±^ males showed a decreased mounting latency and increased intromission frequency and intromission ratio compared with SERT^+/+^ males, suggesting increased sexual motivation and copulatory efficiency, without affecting sexual performance. This is surprising, as differences in aggressive and sexual behavior between SERT^+/+^ and SERT^±^ rodents have not been found before (Chan et al. 2011; Esquivel-Franco et al. 2018; Homberg et al. 2007), and SERT^−/−^ rodents usually show impaired sexual behavior (Chan et al. 2011). When SERT^±^ offspring were split for treatment groups, not many differences were found. In fact, when just looking at SERT^±^ CTR-VEH offspring, which is the most comparable group to previous literature, we only find offensive behavior to be reduced while sexual behavior is unaltered.

Next, we were interested in a possible interaction between offspring SERT genotype and perinatal FLX treatment. In primates, having the short (S) allele for a polymorphism in the promoter region of the SERT gene results in lower SERT expression. Lower SERT expression has been associated with a poorer response to FLX treatment (Stevenson 2018). Keeping this in mind, the reduced SERT expression in our SERT^±^ rats might play a role in the response to perinatal SSRI exposure as well. Interestingly, effects of perinatal FLX treatment on aggressive behavior were primarily observed in SERT^+/+^ offspring and almost completely absent in SERT^±^ offspring. Our study even shows that levels of offensive behavior in SERT^±^ offspring were quite low to begin with, which may have concealed effects of perinatal FLX treatment. When it comes to sexual behavior, perinatal FLX treatment and SERT genotype interacted to decrease mounting in SERT^+/+^ offspring only. Consequently, our findings suggest that SERT^±^ offspring are less sensitive to developmental FLX exposure than SERT^+/+^ offspring. Furthermore, we found an interaction between SERT genotype and ELSD on offspring offensive behavior. In fact, ELSD increased offensive behavior in SERT^±^ offspring only, suggesting that SERT^±^ male offspring are more sensitive to ELSD than SERT^+/+^ offspring. Our results seem in line with the finding that human S-allele carriers that are exposed to stressful life events are more susceptible to stress and have an increased risk to develop mental disorders like major depression. All in all, our results indicate that SERT^±^ offspring are less sensitive to FLX-induced effects on sexual behavior while being more sensitive to ELSD-induced effects on aggressive behavior.

### Limitations and future perspectives

After analyzing our data, we discovered that a large part of our males used for sexual behavior were poor sexual performers. In fact, 46% of our male offspring did not ejaculate at all during the 30-min test session, while sexually experienced SERT^+/+^ and SERT^±^ Wistar rats usually ejaculate on average 2.5 times (Chan et al. 2011). Poor sexual performers were present in all treatment groups. However, frequencies of other copulatory behaviors were within normal range. We also discovered that there were differences in the 4 batches tested. In fact, the 2nd, 3rd, and 4th batches were poor sexual performers, while the 1st batch performed normally. Further analysis did not show an interaction between batch and perinatal FLX treatment, ELSD, or genotype on any of the behavioral parameters tested and indicated a main effect of batch only. Even so, this difference in sexual performance between batches could have concealed effects of FLX, ELSD, or genotype on sexual behavior, which could be present when sexual performance is normal across all batches. Factors responsible for poor sexual performance in these batches might be related to subtle changes during the experiments such as a different season or having a different experimenter that performed testing with each batch (although all data were scored by the same experimenter), but the exact factor remains unknown. Future studies should preferably use one batch of animals, where at least CTR animals should on average display normal sexual performance. Another limitation of the study is that all animals underwent other behavioral tests prior to testing for aggressive and sexual behavior. Being exposed to a behavioral test battery is known to potentially change behavioral outcomes compared with animals naïve for testing experience (McIlwain et al. 2001). The animals used for aggressive behavior were previously exposed to (playful) friendly social interactions at juvenile and adult age, which we believe will have minimal effects on aggressive behavior. Animals used for sexual behavior previously underwent testing for anxiety and depressive-like behavior, which could potentially affect behavioral outcomes. Furthermore, sexual behavior was trained once a week for 7 consecutive weeks. Thus, the behavior reported here is of experienced males, which is comparable with previous studies testing males after several sexual training sessions (Olivier et al. 2011; Rodriguez-Porcel et al. 2011).

Furthermore, the present study investigated male aggressive and sexual behavior only. Previously, it has been shown that effects of developmental FLX exposure can have opposite effects on male and female sexual behavior (Rayen et al. 2013, 2014). Rayen et al. (2013) showed that early postnatal FLX exposure reduces copulatory behaviors in males, while female proceptive and receptive behaviors were facilitated (Rayen et al. 2014). In addition, potential alterations in female aggression, for example, maternal aggression, after perinatal FLX exposure and maternal stress have not been investigated before. Therefore, future research should include both sexes when looking at the effects of perinatal FLX treatment and maternal stress on offspring aggressive and sexual behavior.

## Conclusions

In summary, our research showed that perinatal FLX treatment has profound long-term effects on male aggressive behavior, while having little impact on male sexual behavior. Our results add to the increasing amount of clinical and preclinical literature linking perinatal SSRI treatment to alter neurobehavioral development in the offspring and suggest a role for offspring SERT genotype. However, the underlying maternal depression might also contribute to long-term neurobehavioral alterations in the offspring. The risks and benefits of SSRI use during pregnancy therefore need further investigation, and more studies should include a translational animal model of maternal depression, to provide better insights into the effects in the offspring. Furthermore, future studies should include both male and female offspring to determine implications for behavioral development later in life. Altogether, understanding the effects of SSRI use during pregnancy and the postnatal period can help depressed women to make more informed decisions about the initiation or continuation of antidepressant treatment during pregnancy.

## Abbreviations

CORT: Corticosterone
CTR: Control
ELS: Early life stress
ELSD: Early life stress in dams
FLX: Fluoxetine
G: Gestational day
MS: Maternal separation
PND: Postnatal day
SERT: Serotonin transporter
SSRI: Selective serotonin reuptake inhibitor
VEH: Vehicle
